# ER^-^/PR^+^ breast cancer: from diagnostic discordance to precision therapy

**DOI:** 10.3389/fonc.2026.1961666

**Published:** 2026-10-05

**Authors:** Yuanhao Shao, Meng Zhou, Zhenwei Hu, Bin Wu, Jing Zhou

**Affiliations:** 1 Department of Thyroid and Breast Surgery, Union Hospital, Tongji Medical College, Huazhong, University of Science and Technology, Wuhan, China; 2 Department of Orthopedics, Union Hospital,Tongji Medical College, Huazhong University of Science and Technology, Wuhan, China; 3 Department of Thyroid and Breast Surgery, People’s Hospital of Dongxihu District Wuhan City and Union Dongxihu Hospital, Huazhong University of Science and Technology, Wuhan, China

**Keywords:** diagnostic discordance, endocrine resistance, ER^-^/PR^+^/HER2^-^ breast cancer, non-canonical progesterone receptor signaling, precision oncology

## Abstract

Estrogen receptor-negative/progesterone receptor-positive/human epidermal growth factor receptor 2-negative (ER^-^/PR^+^/HER2^-^)breast cancer is a rare, controversial type that makes up about 0. 3% of all breast cancer cases and does not follow the traditional ER→PR regulatory hierarchy. This review synthesizes the available evidence on the diagnostic evaluation, clinicopathological characteristics, molecular mechanisms, prognosis, and treatment of ER^−^/PR^+^/HER2^−^(ER^-^/PR^+^) breast cancer. Although there are false-negative ER staining results and pre-analytical/analytical factors, a number of clinical and transcriptomic data support the existence of a reproducible ER^-^/PR^+^subgroup with molecular heterogeneity. These tumors often have a high histological grade, elevated Ki-67 expression, TP53 alterations, and basal-like features, and are closer to triple-negative breast cancer than luminal disease. Potential drivers include ER-independent non-canonical PR activation, epigenetic dysregulation, kinase-mediated receptor phosphorylation, membrane-associated progesterone signaling and receptor network reprogramming. Direct subtype-specific evidence remains limited, and the effectiveness of conventional endocrine therapy is unknown. Current guidelines classify these tumors as hormone receptor-positive/HER2-negative, obscuring clinically relevant heterogeneity. Standardized repeat testing and multi-omic characterization are thus required. Future studies should prioritize prospective multicenter cohorts with paired pre-treatment and post-treatment receptor testing, spatial or single-cell characterization, biomarker-guided stratification and adaptive clinical trials. A PR-oriented precision-medicine framework may improve diagnostic classification and help identify therapeutic vulnerabilities in biologically distinct patient subsets. In addition, this rare phenotype may serve as a useful model for investigating lineage plasticity and endocrine resistance.

## 1 Introduction

Molecular subtyping of breast cancer is now a type of personalized medicine, and according to the status of ER, PR and HER2 biomarkers, different treatment plans are usually chosen. Under the above conditions, a fundamental biological axiom has long been held: PR expression is generally considered to be associated with active ER signaling and is often viewed as falling under the traditional ER→PR regulatory pathway (1–3). The above hierarchy of “ER→PR” has helped us understand the biology of hormone receptors better and strengthen the ER-centric model for endocrine therapy. However, the occurrence of ER^-^/PR^+^ breast cancer, especially in the HER2-negative context, is a biologically incongruous and diagnostically ambiguous type that contradicts the traditional ER→PR model (4–6).

The receptor expression pattern does not align with the basic theory; therefore, first, some scholars assumed it was an artifact of immunohistochemistry (IHC) detection (e. g., false ER negativity). Although some ER^-^/PR^+^ cases may be due to technical errors, the accumulation of transcriptomic and clinical data suggests that at least a portion of ER^-^/PR^+^ breast cancers is biologically meaningful. Importantly, the existence of technical or pre-analytical errors does not necessarily exclude the possibility that a subset of ER^-^/PR^+^ tumors represents a biologically meaningful phenotype. Clinical and molecular studies have identified reproducible differences between ER^-^/PR^+^ tumors and conventional ER^+^/PR^+^ or triple-negative tumors, supporting the hypothesis that ER^-^/PR^+^ breast cancer may constitute a distinct, although heterogeneous, biological group (7).Gene expression analysis around the world has identified various changes in transcription, such as differentially expressed genes; at the same time, clinical studies have proposed outcomes that may differ from both classical luminal tumors and TNBC (5). his cognitive evolution from a “technical anomaly” to a “biological reality” marks an advance in our understanding of the disease’s complexity.

The St. Gallen surrogate definitions of intrinsic subtypes are largely built around ER positivity for luminal-like categories. Therefore, ER^-^/PR^+^/HER2^-^ tumors are not readily accommodated within the conventional Luminal A/Luminal B framework (8, 9). The Molecular Taxonomy of Breast Cancer International Consortium (METABRIC) study (10) categorized it under the ER^+^/PR^-^/HER2^-^ group for molecular characterization, while the California Cancer Registry (n=2,794) analyzed it as a separate special subtype. The latest National Comprehensive Cancer Network (NCCN) guidelines (11) discuss this phenotype as part of hormone receptor-positive (HR^+^)/HER2^-^ breast cancer without recognizing it as an independent entity. This classificatory confusion reflects the dilemma of positioning this subtype within the traditional molecular subtyping system.

We propose that the profound significance of ER^-^/PR^+^ breast cancer far exceeds its epidemiological rarity. As a high-value “model system”, it offers a unique opportunity to study two fundamental biological problems in cancer research: first, cancer cell lineage plasticity—how do cancer cells deviate from the rules of their own lineage to maintain and activate an independent PR program while suppressing the ER program? Second, reprogramming of the signaling network: How do cancer cells use non-classical pathways (e. g., kinase phosphorylation, epigenetic modification) to sustain survival and proliferation after the loss of a key master regulator (ER)? A general model for studying adaptive therapeutic resistance in cancer can be provided by the mechanism of “oncogenic receptor reprogramming” in this system. This paper will systematically build a precision framework for ER^-^/PR^+^/HER2^-^ breast cancer that covers diagnosis, mechanism and treatment.

First, we evaluate whether its reproducible epidemiological and clinicopathological features support recognition as a distinct taxonomic category. We will now explore the non-classical PR signaling pathway and epigenetic reprogramming mechanism of its development. Finally, based on its unique molecular vulnerabilities, we have identified the deficiencies in current treatment methods and put forward a plan for the future of precise therapy and clinical research. Our ultimate goal is to build a clinical diagnostic and therapeutic consensus for this long-neglected group of patients and to explore the origins of cancer heterogeneity and resistance in more depth through this model.

## 2 Epidemiology and clinical features

ER^-^/PR^+^/HER2-breast cancer has an extremely low incidence of approximately 0.3% among all breast cancer cases (4). Across population-based and institutional cohorts, ER^-^/PR^+^/HER2^-^ breast cancer are far less frequent than classical Luminal A-like tumors, although the exact prevalence depends on receptor testing criteria and case reassessment (6, 12). The reported prevalence varies substantially according to the thresholds used to define ER and PR positivity. Differences in the percentage of positively stained tumor cells, staining-intensity requirements and the handling of ER-low or borderline cases may result in the inclusion of biologically different tumors within the ER^-^/PR^+^ category. Recent clinical data further emphasize that receptor-positivity thresholds are an important source of epidemiological and clinicopathological heterogeneity in this subtype (13). The frequency of this phenotype may also vary by race, ethnicity, and geographic background, with some studies reporting different racial distributions for ER^-^/PR^+^ tumors compared with ER-positive disease (14, 15). Studies from East Africa have reported a higher proportion of hormone receptor-negative breast cancers, suggesting possible geographic differences in receptor distribution (16). Clinically, ER^-^/PR^+^ tumors have been associated with a distinct phenotype compared with conventional hormone-receptor-positive disease. Reported characteristics include younger age at diagnosis, higher histological grade, increased proliferative activity and a greater frequency of basal-like or aggressive pathological features. Nevertheless, these associations are not entirely consistent across cohorts, partly because of differences in patient selection, receptor-positivity thresholds and the exclusion or inclusion of HER2-positive tumors (15, 17–20). Therefore, the ER^-^/PR^+^ phenotype should be regarded as clinically heterogeneous rather than as a uniformly aggressive entity. In a prospective cohort of young women with invasive breast cancer, high-grade tumors were common; however, grade-specific data for the ER^-^/PR^+^/HER2^-^ subgroup were not separately reported (21). Data from the Shanghai Cancer Center showed that ER^+^/PR^-^/HER2^-^ breast cancer represents an endocrine-resistant subgroup compared with ER^+^/PR^+^/HER2^-^ tumors (22). However, these findings should not be directly extrapolated to ER^-^/PR^+^/HER2^-^ disease. In HER2-positive breast cancer, distinct ER/PR expression patterns have been associated with different clinical outcomes in a real-world neoadjuvant cohort (23). However, because HER2-positive and HER2-negative tumors differ substantially in biology and treatment response, these findings cannot be directly extrapolated to ER^-^/PR^+^/HER2^-^ disease. Direct data on lymph-node metastasis patterns in ER^-^/PR^+^/HER2^-^ breast cancer remain limited.

Compared with ER-positive tumors, ER^-^/PR^+^ tumors tend to show more aggressive pathological features, including higher grade, higher proliferative activity, and more frequent basal-like marker expression, although the exact biomarker profile varies across studies (4, 15, 17). In comparison with ER^+^/PR^+^ cases (24, 25), ER^-^/PR^+^ tumors have higher IHC scores for the proliferation marker Ki-67 and higher Ki-67 mRNA expression. Molecular subtyping analysis has revealed a unique gene expression profile in ER^-^/PR^+^ tumors, with significant upregulation of multiple genes regulated by the SUZ12 repressor (4). Although TP53 alterations are common in ER-negative and basal-like breast cancers, direct evidence defining a subtype-specific mutational landscape for ER^-^/PR^+^/HER2^-^ tumors remains limited. Therefore, genomic features observed in broader ER-negative or HER2-defined subtypes should be extrapolated with caution (21, 26). ER^+^/PR^-^ breast cancer represents a relatively common discordant hormone receptor phenotype and is associated with reduced endocrine responsiveness (22). In contrast, ER^-^/PR^+^ breast cancer is much rarer, and its biology may involve ER-independent or non-canonical PR-related signaling, although this remains incompletely defined (27, 28).

Morphologically and molecularly, ER^-^/PR^+^ breast cancers have been reported to resemble TNBC more closely than classical ER-positive tumors (4, 29). Although ER^-^/PR^+^ breast cancers may share some clinicopathological features with basal-like or triple-negative breast cancer (TNBC), including high histological grade, increased Ki-67 expression and TP53 alterations, their immune microenvironment remains poorly characterized. By contrast, studies of TNBC have demonstrated variable levels of stromal tumor-infiltrating lymphocytes, PD-L1 expression and responses to immune-checkpoint blockade (30). However, these findings cannot be directly extrapolated to ER^-^/PR^+^/HER2^-^ disease, because PR expression and residual steroid-receptor-associated programs may confer distinct biological features. Accordingly, potential mechanisms of immune evasion in ER^-^/PR^+^ tumors may include impaired antigen presentation, dysregulated interferon and NF-κB/JAK–STAT signaling, recruitment of immunosuppressive myeloid cells and activation of inhibitory immune checkpoints (31, 32); nevertheless, these mechanisms remain biologically plausible rather than established subtype-specific characteristics.

The prognostic features of ER^-^/PR^+^/HER2^-^ breast cancer are intermediate. Multivariate analysis has shown that patients with the ER^-^/PR^+^/HER2^+^ subtype have a significantly reduced breast cancer-specific survival rate (hazard ratio [HR]=1.56) (33), while the ER^-^/PR^+^/HER2^-^ subtype has a better prognosis than TNBC (34). However, other studies have found no statistically significant difference in overall survival (OS) between the ER^-^/PR^+^/HER2^-^ and ER^+^/PR^-^ subtypes (p>0.05). Such discrepancies may stem from the heterogeneity of study populations, including variations in the PR expression threshold (1% vs 10% as the cut-off value) and follow-up duration (median follow-up ranging from 60 to 110 months) (35). In a cohort study of 606,025 women (36), the prognosis of Luminal B-like (ER or PR/HER2) subtypes was significantly worse than that of Luminal A-like subtypes, but precise prognostic data for the ER^-^/PR^+^/HER2^-^ subtype still require confirmation in larger sample studies. Chinese cohort studies have suggested that discordant hormone receptor phenotypes may have distinct clinical behavior. ER^+^/PR^-^/HER2^-^ tumors show endocrine-resistant features compared with ER^+^/PR^+^/HER2^-^ tumors (22), while ER^-^/PR^+^/HER2^-^ disease has been separately evaluated in Chinese cohorts and appears to have clinical outcomes distinct from both classical luminal tumors and TNBC (17, 37). Prognostic findings for ER^-^/PR^+^ breast cancer have been inconsistent across studies. Some cohorts have reported outcomes that are less favorable than those of ER^+^/PR^+^ tumors and more closely resemble those of ER^-^/PR^-^/HER2^-^ disease, whereas other analyzes have found that the prognostic difference is attenuated after adjustment for age, tumor grade, stage and treatment. These observations indicate that the prognostic value of the ER^-^/PR^+^ phenotype may be partly mediated by its associated clinicopathological features rather than by receptor discordance alone (20). Accordingly, prognosis should be interpreted in the context of stage, grade, proliferation, HER2 status, treatment received and the method used to define receptor positivity.

In summary, as shown in **Table 1**, the younger age at diagnosis, high-aggressiveness pathological features, and unique intermediate prognosis between Luminal and TNBC subtypes of ER^-^/PR^+^/HER2^-^ breast cancer all point to a biological nature distinct from the classical hormone-driven model. These clinical evidences strongly support that this subtype should not be simply classified into existing categories but regarded as an independent entity driven by “receptor reprogramming”. Therefore, we advocate that ER^-^/PR^+^/HER2^-^ breast cancer should be treated as a separate subtype rather than being merely included in discussions of Luminal or TNBC subtypes.

**Table 1:** Comparison of ER^-^/PR^+^/HER2^-^, ER^+^/PR^+^/HER2^-^, and TNBC.

Feature	ER^-^/PR^+^/HER2^-^	ER^+^/PR^+^/HER2^-^	TNBC
Pathological Characteristics	High-grade tumors, Basal markers ↑, E-cadherin ↓	Low-grade tumors, Luminal markers ↑	Basal-like phenotype, High Ki-67 expression
Molecular Markers	TP53 mutation (30.3%), SUZ12 ↑	Luminal gene expression profile	Burstein subtypes: BLIA, BLIS, MES, LAR (95)
Response to Endocrine Therapy	Controversial (Benefit observed in some patients)	Sensitive (Response rate >70%)	Not applicable
Response to Chemotherapy	Similar to TNBC (Moderate sensitivity)	Lower sensitivity	High sensitivity (but prone to recurrence)
Prognosis	Intermediate (Better than TNBC, worse than Luminal)	Best (5-year survival rate >90%)	Poorest (High metastasis rate)
Unique Mechanisms	Non-canonical PR activation, Epigenetic reprogramming	Classical ER→PR signaling axis	Stem cell characteristics, Immune infiltration

## 3 Diagnostic discordance and pathological verification

Importantly, ER^-^/PR^+^ status should not be interpreted as a uniform monoclonal phenotype throughout the tumor. It may represent an instantaneous, sample-dependent classification resulting from the coexistence of biologically distinct subclones, including basal-like/ER-negative and luminal-like cell populations. Single-cell and spatial transcriptomic studies have demonstrated substantial intratumoral diversity and the coexistence of luminal, basal-like and intermediate cell states in breast cancer (38–40).These findings provide a plausible framework in which a TNBC-like subclone may acquire PR-associated transcriptional activity or a luminal subclone may lose ER expression; however, direct single-cell evidence demonstrating these trajectories specifically in ER^-^/PR^+^/HER2^-^ tumors remains limited. Bulk IHC and conventional core-biopsy sampling may therefore obscure spatially restricted or subclonal receptor discordance. Heterogeneity may also vary according to PR expression level: tumors with PR expression in 1%–10% of invasive tumor cells may be more susceptible to sampling variation, technical variability and subclonal interpretation than tumors with >10% expression. Nevertheless, the 10% cutoff has not been validated as a biological or treatment-predictive threshold in ER^-^/PR^+^/HER2^-^ disease, and both groups should be investigated separately rather than treated as homogeneous entities (3).

## 4 Molecular biological mechanisms

In the classical model, PR is regarded as a downstream effector of ER signaling, and its expression is strictly dependent on ER activity. However, the existence of the ER^-^/PR^+^ phenotype forces us to address a core question: how is PR driven and sustained to exert its oncogenic function in the absence of ER, the master regulator? This section aims to systematically demonstrate that this phenotype is not a technical artifact but driven by a set of ligand-independent, kinase- and epigenetic-dominated “oncogenic receptor reprogramming” mechanisms. This reprogramming transforms PR from a downstream effector of ER into an independent tumor-driving engine.

### 4.1 Potential onset of ER silencing and PR retention

Beyond the question of how PR is activated in the absence of ER lies a more fundamental issue: what allows this paradoxical receptor profile to emerge in the first place? Several lines of evidence point to upstream events that may create a permissive environment for ER-independent PR expression.

Growth factor signaling represents one such mechanism. There is a crosstalk between growth factors and steroid receptors: epidermal growth factor (EGF) and insulin-like growth factor 1 (IGF-1) have been confirmed to activate PR expression in an ER-independent manner. Borras first verified this phenomenon by establishing the Evsa-T human breast cancer cell line (ER^-^/PR^+^) (27). In addition, in cancer tissues, PR may be activated in the absence of classical ligands or at low ligand concentrations to form abnormally activated PR (APR), a process mediated by growth hormone, possibly through CK2-mediated Ser81 phosphorylation and subsequent PR activation (28, 41).

Another factor potentially associated with ER-independent PR expression is the high mobility group A1 (HMGA1) protein. Studies have shown a positive correlation between HMGA1 overexpression and PR expression, and a negative correlation with ER expression. In breast cancer, HMGA1 mainly affects endocrine therapy response by regulating the miR-181b/CBX7 pathway (42). A study predicting breast cancer receptor expression status by microRNAs (miRNAs) indicated that several miRNAs (miR-520g, miR-377, miR-527-518a, miR-520f-520c) can predict PR expression status (43), suggesting that HMGA1 may regulate PR expression by modulating miRNA profiles. Sex hormone splice variants may also be involved in hormone receptor expression. In all patients, ERβΔ5 mRNA expression is significantly negatively correlated with PR protein levels; in postmenopausal patients, ERβΔ5 is negatively correlated with both PR and ERα (44).

These upstream factors likely represent initiating events that disrupt the classical ER-PR hierarchy, creating conditions permissive for the emergence of this unusual phenotype. However, they should be distinguished from the core driving mechanisms that sustain tumor progression once the reprogrammed state is established, which we discuss in the following sections.

### 4.2 Non-classical activation of PR and oncogenic signaling network

The core driving force for the progression of ER^-^/PR^+^/HER2^-^ breast cancer lies in the non-classical activation of PR and the remodeling of the oncogenic signaling network it mediates in the context of ER deficiency. Based on differences in core driving pathways, this subtype can be divided into two subgroups: kinase phosphorylation-driven and progesterone receptor membrane component 1 (PGRMC1) membrane signaling axis-driven. Both achieve ligand-independent activation of PR and mediate tumor proliferation, invasion, and metastasis, and may exist in a co-activated or single-activated state in the same tumor, contributing to intratumoral and intertumoral heterogeneity of this subtype.

#### 4.2.1 Kinase phosphorylation-driven transcriptional reprogramming of PR

PR mainly exists in two isoforms: PRA and PRB, which differ in structure and thus function. The PRA/PRB ratio may influence metastatic behavior in breast cancer, with a high PRA ratio associated with a more aggressive phenotype, although the specific regulatory pathways remain unclear (45). In ER^+^/PR^+^/HER2^-^ breast cancer, PR can also act as a genomic agonist in an isolated state to regulate the expression of common target genes without complete dependence on ER. This indicates that PR has intrinsic transcriptional regulatory capacity and can affect tumor growth even in an ER-free environment (46). Further studies have found that in an ER-negative context, kinases such as mitogen-activated protein kinase (MAPK/ERK1/2) can phosphorylate specific serine residues of PR (e.g., Ser81, Ser294) to activate the receptor. Phosphorylated PR (p-PR) acquires ligand-independent transcriptional activity and can form a complex with kinases such as ERK1/2 and mitogen- and stress-activated kinase 1 (MSK1), which is directly recruited to transcriptional repressor genes involved in cell proliferation (28, 41, 47). This activation mechanism differs from the traditional ligand-dependent pathway and involves the synergy of the BRG1 and HP1γ-LSD1 repressive complexes, anchored by SUV39H2-mediated H3K9me3 epigenetic marks to regulate tumor growth and proliferation (47). Activated MAPK and other pathways not only phosphorylate PR but may also be amplified by the transcriptional output of PR, forming a positive feedback regulatory loop that stabilizes this non-classical activated state.

Meanwhile, p-PR can also act on ER/PR target genes through its interaction with insulin receptor substrate-1 (IRS-1), promoting endocrine resistance and the growth of breast cancer stem cells (48). Notably, the 16-gene signature marked by Ser294-phosphorylated PR (p-PR) can predict poor prognosis, further validating the central role of kinase-mediated PR modification in disease progression. Kinase inhibition experiments (49) have confirmed that combined blockade of phosphoinositide 3-kinase (PI3K) and fibroblast growth factor receptor 4 (FGFR4) can synergistically inhibit the proliferation of ER^-^/PR^+^/HER2^-^ tumor cells.

In addition, PR can interact with interferon (IFN) or immune signaling pathways independent of ER. For example, PR can inhibit type I IFN signaling (e.g., IFNα/β) by promoting the ubiquitination and degradation of signal transducer and activator of transcription 2 (STAT2), thereby helping tumor cells evade immune surveillance. This mechanism has been confirmed in breast cancer cells, where PR expression directly blocks the interferon signaling cascade without ER involvement (50).

#### 4.2.2 Synergistic driving by the PGRMC1 signaling axis

PGRMC1 is an important non-classical progesterone-binding protein, which has been confirmed to be associated with lymph node metastasis, larger tumor size, and poor overall and tumor-free survival (51). In breast cancer, the prognostic value of PGRMC1 is independent of ER status, meaning that high PGRMC1 expression may still be associated with a poor prognosis in ER-negative subtypes (52).

When PGRMC1 is expressed in breast cancer tissues, it can enhance the pro-proliferative effect of progestogens, i.e., PGRMC1 may drive tumor growth by amplifying the progesterone signaling pathway (53). Beyond this tandem effect with PR, its core role is to provide a rapid, non-genomic pro-survival and pro-metastatic signaling pathway. In ER^-^/PR^+^/HER2^-^ breast cancer, PGRMC1 expression may provide an independent pro-growth signaling axis. Studies have shown that PGRMC1 can promote breast cancer growth by enhancing the epidermal growth factor receptor (EGFR)/PI3K/AKT/mammalian target of rapamycin (mTOR) signaling axis and inhibiting phosphatase and tensin homolog (PTEN) expression (54). In addition, elevated PGRMC1 expression may activate the Janus kinase 2 (JAK2)/STAT3 pathway to promote epithelial-mesenchymal transition (EMT) (55), a process independent of classical nuclear PR signaling. Research on the expression and function of PGRMC1 in ER^-^/PR^+^/HER2^-^ breast cancer is an important direction to reveal the poor prognosis of this subtype.

Non-canonical PR activation alone, however, may not fully explain how ER^-^/PR^+^ tumor cells maintain proliferation and survival after loss of ER. This phenotype should therefore be considered in the context of broader pathway crosstalk.

### 4.3 ER-loss-driven pathway crosstalk and survival adaptation

ER loss in ER^-^/PR^+^ breast cancer is unlikely to be an isolated receptor event and may instead mark a transition from an ER-dominant transcriptional state to a compensatory, network-dependent state. Clinical studies indicate that at least a subset of ER^-^/PR^+^ tumors displays high histological grade, elevated proliferative activity and clinicopathological or treatment-response characteristics that differ from those of conventional ER^+^/PR^+^ tumors, supporting the rationale for investigating ER-independent proliferative mechanisms in this phenotype (7, 56). Persistent PR expression may constitute one component of this adaptive state. However, because direct pathway-level studies in ER^-^/PR^+^ tumors remain scarce, the mechanisms discussed below should be interpreted as a biologically supported model that integrates ER^-^/PR^+^ clinical observations with established evidence from PR biology and endocrine-resistant breast cancer, rather than as molecular features already demonstrated in all ER^-^/PR^+^ tumors.

Metabolic reprogramming, inflammatory signaling and apoptosis avoidance may represent downstream components of the same adaptive network rather than independent defining mechanisms. PI3K/AKT/mTOR and MAPK activation can increase nutrient uptake, glycolytic and lipid-biosynthetic capacity, protein translation and oxidative-stress tolerance, providing the energetic and biosynthetic support required for sustained proliferation (57). In parallel, NF-κB- and JAK/STAT-related inflammatory programs can enhance cellular plasticity, stress tolerance and interaction with the tumor microenvironment, while PI3K/AKT and anti-apoptotic BCL-2-family signaling can suppress therapy- or stress-induced cell death (58). A plausible integrated model is therefore that, following ER loss, PR-associated transcription preserves part of the steroid-receptor program; receptor tyrosine kinases and PI3K/AKT/mTOR provide proliferative and survival signals; metabolic adaptation supports biosynthetic demands; and inflammatory and anti-apoptotic pathways promote plasticity and viability. Functional redundancy among these pathways could allow a subset of ER^-^/PR^+^ tumors to survive despite loss of ER and could also limit the efficacy of targeting PR or any single pathway alone (59). Importantly, direct evidence that these metabolic, inflammatory and apoptotic programs are selectively activated in ER^-^/PR^+^ breast cancer is currently insufficient. This model should therefore be tested using centrally confirmed ER^-^/PR^+^ specimens, phosphoproteomic profiling, metabolomics and functional perturbation studies.

In addition to these signaling adaptations, epigenetic remodeling may stabilize the ER-negative/PR-positive cell state by suppressing the ER transcriptional program while preserving selected PR-associated and kinase-responsive regulatory networks.

### 4.4 Epigenetic-related mechanisms

The two major reprogramming mechanisms described above are not transient states but are “memorized” and solidified through profound epigenetic remodeling, ensuring the stable and highly aggressive biological phenotype of this subtype. Differences in DNA methylation patterns are the key mechanism leading to ER silencing with retained PR expression, and the methylation status of ESR1 plays an important role in the ER-negative tumor phenotype (60). Studies have found the upregulation of SUZ12-regulated genes in ER^-^/PR^+^ breast cancer, suggesting a critical role of polycomb repressive complex 2 (PRC2) in this subtype (4). SUZ12 is a core component of PRC2, a major epigenetic regulatory complex that mediates gene silencing by catalyzing the trimethylation of histone H3 at lysine 27 (H3K27me3) and is associated with the development of various cancers, including breast cancer (61).

In addition, other epigenetic mechanisms may also be involved in the development of this subtype. Indoleamine 2,3-dioxygenase 1 (IDO1), a key enzyme in tryptophan catabolism, mainly functions in tumor immune evasion. In ER^+^ breast cancer, the methylation of the IDO1 promoter inhibits its expression, while this methylation is reduced in ER^-^ breast cancer, leading to increased IDO1 expression and thus enhanced cancer cell invasiveness (62). Steroid receptor coactivator 1 (SRC-1), an endoplasmic reticulum regulator, can mediate gene silencing in a genome-wide epigenetic manner, which inhibits cell differentiation (63). However, the role of SRC-1 in breast cancer has been mainly studied in ER^+^ subtypes, with a lack of targeted research on ER^-^/PR^+^ breast cancer. Given its potential involvement in a broad range of steroid receptor signaling pathways, including PR, future research can focus on exploring the role of SRC-1 in a PR-positive environment to fill this gap. Glucocorticoid receptor (GR) activation in ER^+^ breast cancer leads to a decrease in histone acetylation (H3K20Ac) levels at proliferation-related gene loci, promoting chromatin condensation and inhibiting breast cancer cell metastasis. In ER-negative breast cancer cells, GR exhibits a pro-tumor metastatic effect (64);although the specific oncogenic mechanism remains unclear, it is speculated to be associated with epigenetic remodeling.

Based on the above, we propose a potential multi-level dynamic reprogramming process underlying the development and progression of ER^-^/PR^+^/HER2- breast cancer (**Figure 1**). Upstream transcriptional/epigenetic inducers may initiate the abnormal pattern of ER silencing and PR retention; kinase phosphorylation-driven nuclear PR transcriptional reprogramming and PGRMC1-mediated membrane signaling axes take over the function of driving tumor proliferation, survival, and metastasis; these two pathways can further influence the PRC2-dominated epigenetic regulatory network through downstream effects (e.g., recruiting chromatin regulators, activating specific kinases); the solidified epigenetic landscape, in turn, determines the regulatory efficiency of kinase pathways on PR and the expression of PGRMC1-related genes, forming a self-sustaining and self-reinforcing oncogenic cycle. This integrated molecular regulatory network perfectly explains the unique transcriptomic signature, TNBC-like clinicopathological features, and intrinsic resistance to conventional endocrine therapy of this subtype, while molecular differences between subgroups provide core targets for precision therapy.

**Figure 1:**
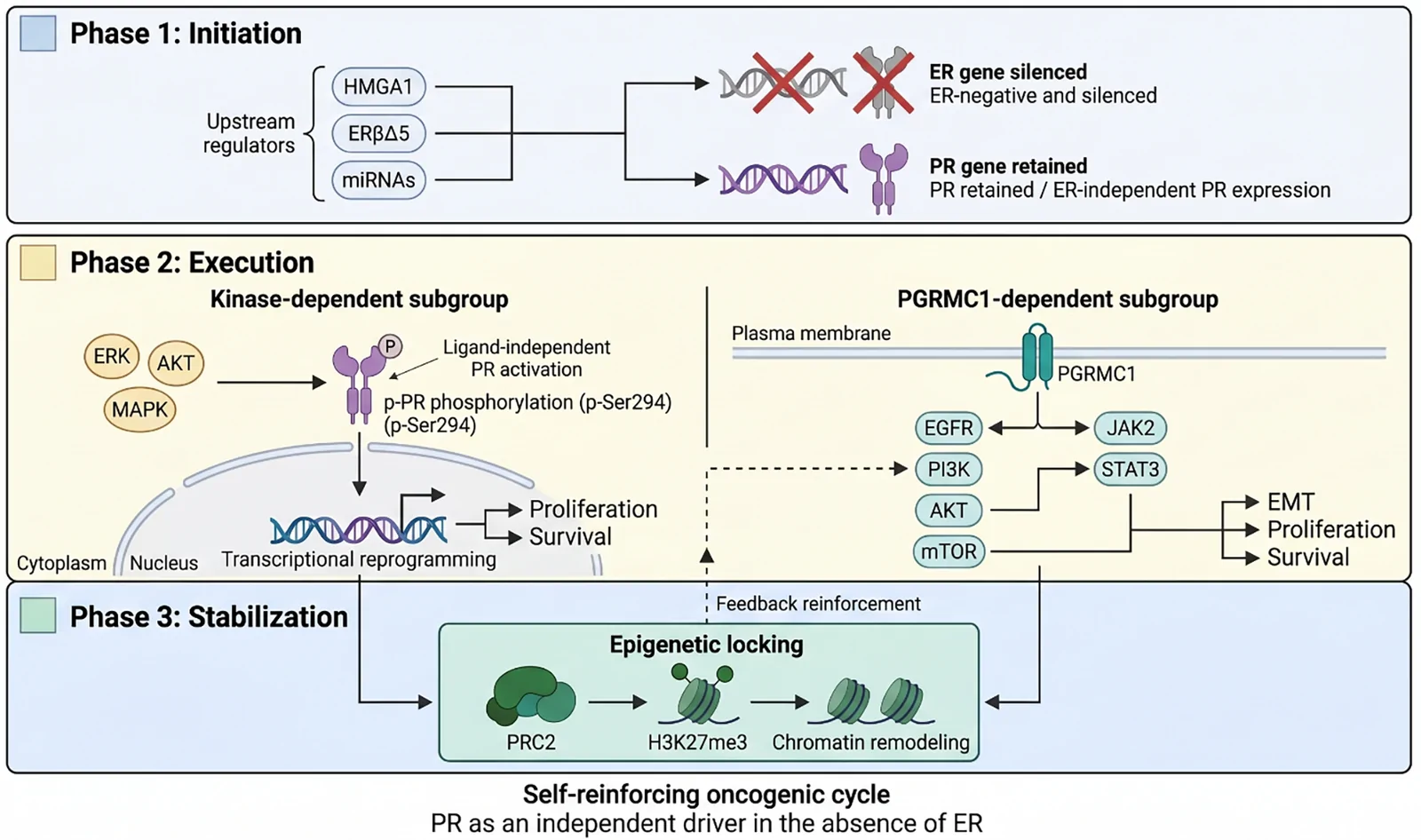
Three-phase model of oncogenic PR reprogramming in ER^-^/PR^+^/HER2^-^ breast cancer. The model illustrates the dynamic process through which PR becomes an independent driver in the absence of ER. Phase 1 (Initiation): Upstream factors including HMGA1, specific miRNAs, CK2, and ERβΔ5 create a permissive environment for ER silencing and PR retention. Phase 2 (Execution): Two distinct subgroups drive tumor progression. The kinase-driven subgroup (left) involves Ras/Raf/ERK-mediated phosphorylation of PR (p-Ser294), enabling ligand-independent transcriptional activity. The PGRMC1-driven subgroup (right) involves membrane-initiated signaling through EGFR/PI3K/AKT/mTOR and JAK2/STAT3 pathways, promoting proliferation, EMT, and survival. Phase 3 (Stabilization): Both pathways converge on PRC2-mediated epigenetic remodeling (H3K27me3), which locks the re programed state and maintains gene expression patterns. A feedback loop from the stabilized chromatin state reinforces the activity of the execution pathways, forming a self-sustaining oncogenic cycle. ER: estrogen receptor; PR: progesterone receptor; PGRMC1: progesterone receptor membrane component 1; PRC2: polycomb repressive complex 2; EMT: epithelial-mesenchymal transition. Created in BioRender. Shao, Y. (2026) https://BioRender.com/ipt3vw2.

## 5 Treatment strategies and challenges

### 5.1 Current treatment strategies and limitations

The current primary systemic treatment modalities for ER^-^/PR^+^/HER2^-^ breast cancer are chemotherapy and endocrine therapy (65). Because the clinical and molecular characteristics of ER^-^/PR^+^/HER2^-^ breast cancer are relatively close to those of TNBC, chemotherapy regimens similar to those for TNBC are often selected and can achieve certain efficacy (29, 56). However, the response of ER^-^/PR^+^/HER2^-^ breast cancer to endocrine therapy is highly controversial. The latest NCCN guidelines (11) state that endocrine therapy may be considered for ER^-^/PR^+^/HER2^-^ breast cancer, but efficacy data are limited. Evidence shows (22) that ER^+^/PR^-^/HER2^-^ breast cancer has lower responsiveness to endocrine therapy compared to ER^+^/PR^+^/HER2^-^ breast cancer, which might be related to the absence of the PR receptor; therefore, it can be inferred that in an ER-negative background, isolated PR positivity might be insufficient to drive an endocrine therapy response. However, paradoxical phenomena have also been observed: some patients with ER^-^/PR^+^ tumors still benefit from anti-estrogen therapy. This heterogeneity might be associated with low-level expression of estrogen receptors in the tumor microenvironment or limitations in detection techniques. A retrospective analysis of breast cancer patients at the Cancer Hospital, Chinese Academy of Medical Sciences (66) showed that ER^-^/PR^+^/HER2^-^ patients receiving adjuvant endocrine therapy had better relapse-free survival (RFS) and overall survival (OS) than those who did not receive endocrine therapy. Similarly, a prospective cohort study from China (37) also indicated that postmenopausal and age ≥55 years ER^-^/PR^+^ patients could benefit from endocrine therapy. Research into novel treatment approaches is urgently needed to meet clinical demands.

Clinical practice should begin with standardized pathological confirmation, which is the prerequisite for all subsequent therapeutic decisions, followed by individualized management. ER, PR and HER2 testing should follow current ASCO/CAP standards, including assessment of specimen adequacy, fixation, internal controls, staining intensity and the percentage of positive invasive tumor cells. Therefore, unexpected ER^-^/PR^+^/HER2^-^ results should be verified by review of specimen adequacy and internal controls, repeat IHC on another block or biopsy when feasible, and multidisciplinary pathological review. When surgery is subsequently performed, receptor testing on the resection specimen should be considered if the initial result is discordant, technically limited or clinically inconsistent (3, 67). Conventional PR positivity should be based on unequivocal nuclear staining in invasive tumor cells; isolated cytoplasmic or membranous staining should be reported separately and should not be counted as conventional PR positivity. These tumors should not automatically be treated as either conventional luminal or triple-negative breast cancer. Treatment decisions should instead be discussed by the breast multidisciplinary team (MDT), integrating tumor stage, histological grade, Ki-67, genomic features, patient age and comorbidities (68). Endocrine therapy may still be considered when repeat testing demonstrates ER-low or heterogeneous ER expression, a convincing luminal biology, or when chemotherapy is unsuitable; however, its benefit should not be assumed to be equivalent to that observed in conventional ER-positive luminal tumors, and PR positivity alone is insufficient to establish endocrine sensitivity (69). Conversely, PR positivity alone should not justify omission of systemic chemotherapy in tumors with high-risk or basal-like features. The pathology report should clearly flag the ER^-^/PR^+^/HER2^-^ phenotype and any interpretive uncertainty, with direct pathologist–clinical team communication maintained throughout diagnosis, staging, treatment selection, response assessment and recurrence management.

After pathological confirmation, neoadjuvant chemotherapy may be considered for medically fit patients with stage II–III disease, high histological grade, high Ki-67, basal-like features, or other high-risk characteristics, whereas endocrine therapy alone should not be routinely used for a truly ER-negative tumor. Endocrine therapy, including an aromatase inhibitor with ovarian suppression in selected premenopausal patients or an aromatase inhibitor in postmenopausal patients, may be considered only when repeat testing identifies ER-low or heterogeneous ER expression, a convincing luminal biology, or when chemotherapy is unsuitable (70); its benefit in true ER^-^/PR^+^ disease remains unproven.

For tumors reported as ER^-^/PR^+^ after neoadjuvant therapy, treatment should be based on a comparison of pre-treatment biopsy and post-treatment residual disease rather than on the post-treatment result alone. The residual tumor should undergo repeat ER, PR and HER2 assessment, with review of staining distribution and technical adequacy (65, 71). If the patient has completed the planned neoadjuvant chemotherapy, a new chemotherapy course should not be initiated solely on the basis of receptor conversion; subsequent therapy should instead be guided by residual disease burden, treatment response, baseline and residual tumor biology, germline BRCA1/2 status and other validated risk factors (72). Capecitabine may be considered for selected patients with residual high-risk HER2-negative disease, although this recommendation is extrapolated primarily from TNBC evidence (73), whereas adjuvant olaparib should be reserved for patients meeting high-risk criteria for germline BRCA1/2-mutated, HER2-negative early breast cancer (74). Endocrine therapy should be continued or initiated when ER positivity is confirmed before or after treatment, particularly when expression is heterogeneous or ER-low; however, the benefit of endocrine therapy in ER-low disease remains uncertain, and isolated post-treatment PR positivity in the absence of ER expression should not automatically mandate endocrine therapy (70).

### 5.2 Mechanism-guided targeted treatment framework

To break through this dilemma, a precision therapeutic framework directly linked to molecular mechanisms must be established, and future treatment options should be based on the analysis of the dominant driving mechanisms of tumors. The major candidate biomarkers, therapeutic targets, and translational strategies are summarized in **Table 2**. For this type of breast cancer, in addition to conventional chemotherapy or endocrine therapy regimens, PR antagonists or selective progesterone receptor modulators (SPRMs) should be used as first-line therapy. Mifepristone and 12-O-tetradecanoylphorbol-13-acetate (TPA) have been confirmed in animal model experiments to inhibit progesterone-induced oncogenic pathways, suggesting the potential of PR antagonists in breast cancer treatment (75). Further studies are needed to evaluate their efficacy and safety in clinical applications. Combined targeted therapy based on PR antagonists represents a more promising therapeutic strategy. For patients with high Ser294 PR expression, combination therapy with phosphorylation pathway-related inhibitors may be considered. For ER^+^ breast cancer, several related combination regimens have been validated in clinical trials, such as alpelisib (PI3Kα inhibitor) plus fulvestrant (76) or capivasertib (pan-AKT inhibitor) plus fulvestrant (77). Unfortunately, no such combination regimens have been used in clinical or preclinical studies for PR-single-positive breast cancer. For breast cancer subtypes with PGRMC1 expression, combination therapy with PGRMC1 antagonists should be administered. Currently, there are no clinically approved specific PGRMC1 antagonists, but some compounds (e.g., AG-205) have shown potential for targeting PGRMC1 in preclinical studies (78).

**Table 2:** Potential precision treatment strategies framework for ER^-^/PR^+^/HER2^-^ breast cancer.

Mechanism subtype	Predictive biomarker	Potential targeted therapy strategy
Kinase Phosphorylation-Driven	High p-Ser81/294 PR expression (IHC)	SPRM (e.g. Mifepristone) + MEK inhibitor (e.g. Selumetinib) or PI3K inhibitor (e.g. Alpelisib)
PGRMC1 Signaling Axis-Active	High-PGRMC1 expression (IHC)	SPRM (e.g. Mifepristone) + PGRMC1 inhibitor (e.g. AG-205)
Genomic Instability	TP53 mutation	SPRM (e.g. Mifepristone) + PARP inhibitor (e.g. Olaparibv)

Genomic analyzes reveal unique molecular vulnerabilities in ER^-^/PR^+^/HER2^-^ breast cancer. These tumors exhibit high genomic instability, with 30.3% of cases harboring TP53 mutations, significantly higher than the ER^+^/PR^+^/HER2^-^ subtype (10.1%) (22). This mutation profile suggests that drugs targeting DNA damage repair, such as PARP inhibitors, might have therapeutic potential. Furthermore, tumors that are HER2-negative but express the p95HER2 truncated variant are insensitive to conventional HER2-targeted therapies, while antibody-drug conjugates (ADCs) targeting p95HER2 or immunotherapeutic agents might become effective alternatives (79). Multi-omics analyzes have also identified subtype-specific fusion genes and copy number variations, providing a molecular basis for developing personalized targeted treatment regimens (80).

## 6 Future research directions

Based on the above discussion, we believe that future research on ER^-^/PR^+^/HER2^-^ breast cancer should prioritize deeper characterization of tumor heterogeneity, identification of actionable therapeutic targets, and development of a standardized diagnostic and treatment framework.

### 6.1 Model construction and heterogeneity resolution

Existing studies have shown that ER^-^/PR^+^ breast cancer has a unique transcriptomic signature, distinct from both typical ER^+^ tumors and TNBC. Future research can conduct large-sample single-cell/spatial multi-omics studies on ER^-^/PR^+^/HER2^-^ breast cancer patients to verify the clinical universality of kinase/PGRMC1-driven subgroups and explore subgroup-specific molecular biomarkers and regulatory pathways. In addition to subclonal diversity and PR-expression-level variation, androgen receptor (AR) expression may introduce an additional layer of biological heterogeneity in ER^-^/PR^+^/HER2^-^ breast cancer (81). AR and PR are closely related steroid hormone receptors that share transcriptional cofactors and may engage in overlapping regulatory networks, although their functional interplay is highly context dependent. In ER-negative breast cancer, AR expression is a hallmark of the luminal androgen receptor (LAR) molecular subtype and may identify tumors with a more luminal-like, androgen-driven biology; however, AR positivity alone does not define luminal lineage or predict endocrine sensitivity (82). Consequently, co-expression of AR and PR might represent an alternative hormone-receptor program operative in a subset of ER^-^/PR^+^ tumors, whereas AR-negative/PR-positive tumors may reflect a distinct, potentially more basal-like or non-canonical PR-driven state. Nevertheless, direct evidence linking AR–PR co-expression to the clinical definition or treatment response of ER^-^/PR^+^/HER2^-^ breast cancer remains scarce. Therefore, AR should be considered primarily as a stratification biomarker for biological heterogeneity rather than as a defining criterion for this subtype.

Combined with single-cell CRISPR screening technology (83, 84), the core regulatory factors involved in subgroup formation can be identified to provide a basis for the development of new targeted targets. Meanwhile, the interaction mechanisms between subgroups and cancer stem cells, as well as the immune microenvironment, should be explored to provide new ideas for combined immunotherapy. In addition, the PDO culture system for ER^-^/PR^+^ breast cancer needs to be optimized, and tumor microenvironment organoid models (incorporating immune cells and stromal cells) should be constructed to better simulate the clinical tumor microenvironment (85). Based on the PDO model library, high-throughput drug screening should be carried out to identify novel targeted drugs and combination regimens for kinase/PGRMC1-driven subgroups. At the same time, drug-resistant models of PDOs should be established to analyze subgroup-specific resistance mechanisms, providing a basis for overcoming therapeutic resistance (86).

### 6.2 Innovation of diagnostic techniques and construction of AI models

Current diagnosis of ER^-^/PR^+^ breast cancer is plagued by significant technical controversies, with the reported incidence varying by up to 10-fold (0.3%-3%) across different research centers. International collaborative groups are in urgent need of establishing standardized detection protocols, including unified antibody clone numbers, antigen retrieval methods, and positive threshold settings. The subcellular localization of PR (nuclear/cytoplasmic distribution) is associated with different functions and mechanisms, which may facilitate further precise subtyping of PR-single-positive breast cancer and guide its treatment (55, 87). Future validation studies based on multicenter cohorts are required to evaluate the clinical significance of PR subcellular localization. The application value of novel molecular imaging technologies such as Ga-NeoB positron emission tomography/computed tomography (PET/CT) in receptor detection also needs prospective validation, which can provide functional supplementation for traditional IHC (88). In addition, artificial intelligence (AI) can be applied to breast cancer diagnosis: for example, AI-assisted diagnosis (AI-CAD) in screening mammography can identify cancer features missed by radiologists (89) and can also be used to predict cancer biological characteristics and clinical outcomes (90). Previous research has evaluated the use of AI for histopathologic classification and ER-status assessment in breast cancer (91); thus, the AI prediction model should be continuously constructed and optimized to improve its accuracy and generalization ability. Meanwhile, a visual interface and clinical reporting system for the AI model should be developed to facilitate its use by clinicians and realize intelligent clinical decision support.

### 6.3 Validation of therapeutic strategies and construction of diagnosis and treatment systems

From a translational perspective, p-PR and PGRMC1 are promising candidate biomarkers because they may capture PR activation and membrane-associated progesterone signaling that are not reflected by total PR immunoreactivity alone (69). However, neither biomarker is currently sufficiently validated for routine treatment selection in ER^-^/PR^+^/HER2^-^ breast cancer (3). Standardized assays, predefined positivity thresholds and prospective correlation with response and survival should therefore be incorporated into clinical validation studies.

Umbrella trials are a classic design for biomarker-guided precision oncology clinical research (92). Given the biological heterogeneity suggested by current data, biomarker-guided umbrella trial designs may be worth exploring in carefully characterized ER^-^/PR^+^/HER2^-^ cohorts. Patients should be assigned to corresponding targeted therapy substudies based on different biomarkers (e.g., p-PR, PGRMC1, TP53 status) to validate the above precision therapeutic strategies and accumulate high-level evidence-based medical evidence. Adaptive clinical trial designs (93) should be incorporated to enable strategy refinement on the basis of interim findings and to facilitate more efficient identification of effective regimens; liquid biopsy approaches, particularly circulating tumor DNA (ctDNA) (94), may provide a practical means for dynamic biomarker monitoring and longitudinal response assessment. If future prospective studies validate reproducible molecular subgroups and actionable targets, the development of subtype-specific expert recommendations may become feasible.

## 7 Conclusion

In conclusion, ER^−^/PR^+^breast cancer is a rare and clinically heterogeneous phenotype that cannot be fully explained by technical artifacts or accommodated within conventional luminal categories. Although standardized repeat testing is essential to exclude false-negative ER results and sampling-related discordance, accumulating clinical and molecular evidence suggests that at least a subset of these tumors may represent a biologically meaningful group. Their heterogeneity may reflect the coexistence of distinct cellular states, noncanonical PR signaling, epigenetic dysregulation, kinase-mediated receptor activation, and alternative hormone-receptor programs, including potential AR-related subgroups. However, the direct evidence supporting these mechanisms and their treatment implications remains limited. Therefore, ER^−^/PR^+^status should not be used alone to define prognosis or endocrine sensitivity. Future prospective multicenter studies incorporating repeated receptor testing, multi-omic, spatial, and single-cell analyses, as well as biomarker-guided clinical trials, are needed to refine diagnostic classification and identify effective precision-treatment strategies.
